# Supplementary material for: Comparative Transcriptome and Methylome Analysis in Human Skeletal Muscle Anabolism, Hypertrophy and Epigenetic Memory
Source: Sci Rep. 2019 Mar 12;9:4251. doi: 10.1038/s41598-019-40787-0 (PMC6414679; doi:10.1038/s41598-019-40787-0)
Supplement: Supplementary file 1 — Suppl. File 1 [file 41598_2019_40787_MOESM1_ESM.pdf]

Title: Comparative Transcriptome and Methylome Analysis in Human Skeletal Muscle Anabolism, Hypertrophy and Epigenetic Memory

Authors: Daniel C. Turner, Robert A. Seaborne, Adam P. Sharples*

*Corresponding Author

Suppl. Figure 1 & 2

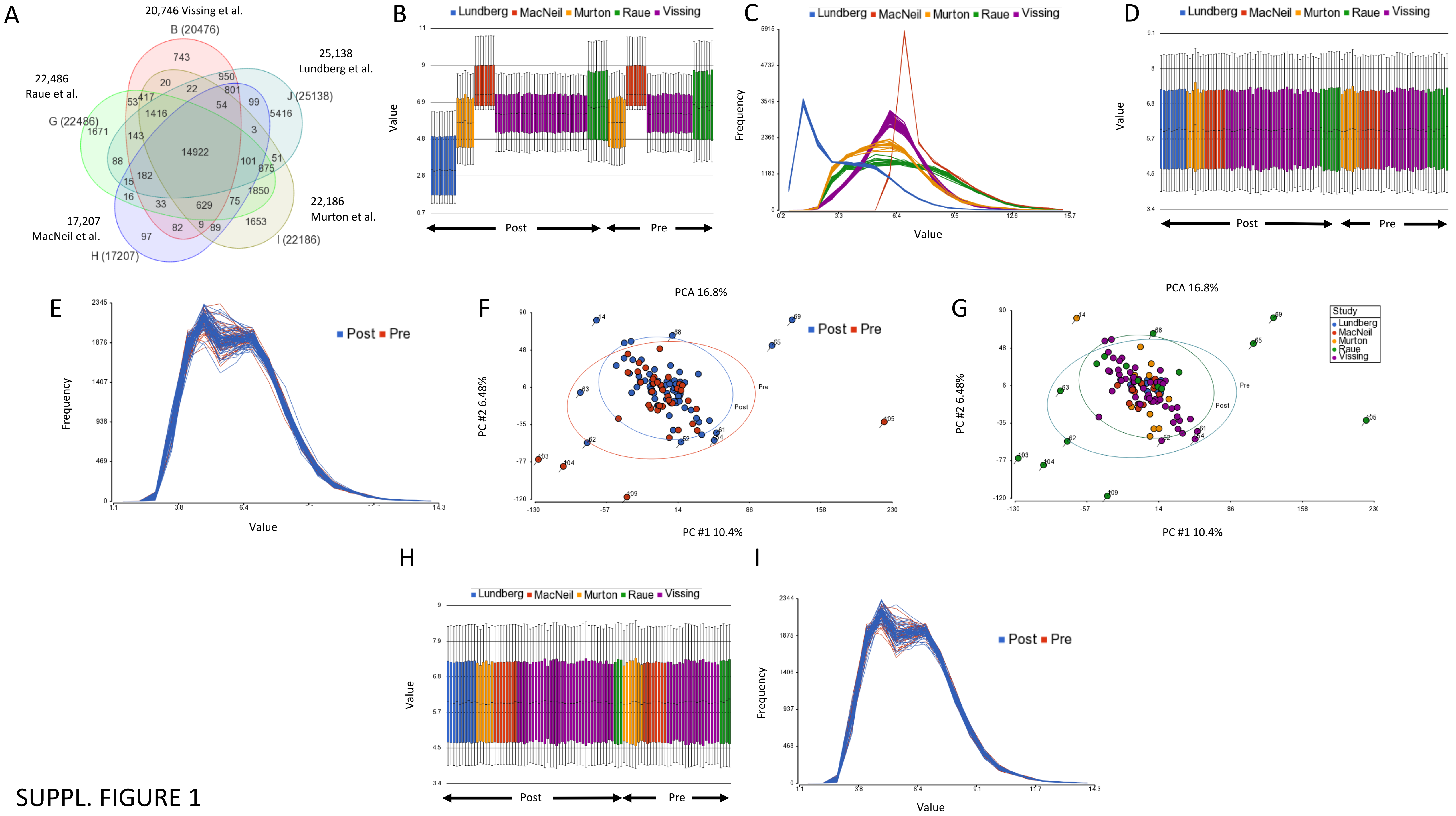

SUPPL. FIGURE 1

Suppl. Figure 1: **A**, Venn diagram analysis identified a shared list of 14,992 annotated genes by ‘gene symbol’ across the pooled transcriptomic studies for acute RE. Note [4,5] use the same gene array platform but the number of genes is depicted as different. This is due to the highest probe set analysis (see methods) altering the genes that could be compared in this study. Inspecting **B**, box and whisker plots before batch correction and, **C,** frequency plots by lines there was a noticeable signal/ value variation as a result of batch effects across studies. **D,** Box and Whisker plots post batch correction and **E,** frequency normalisation plots by lines post batch correction**. F,** PCA post batch correction by time (pre/post) and **G:** PCA by study depicting the outliers removed (line strikethrough) that were located outside 2SD’s of the centroid value using ellipsoids. **H,** Sample box and whiskers plot with batch correction and outlier samples (highlighted in F/G) removed. **I,** Frequency Plot by Lines after batch correction and with outlier samples (highlighted in F/E) removed.

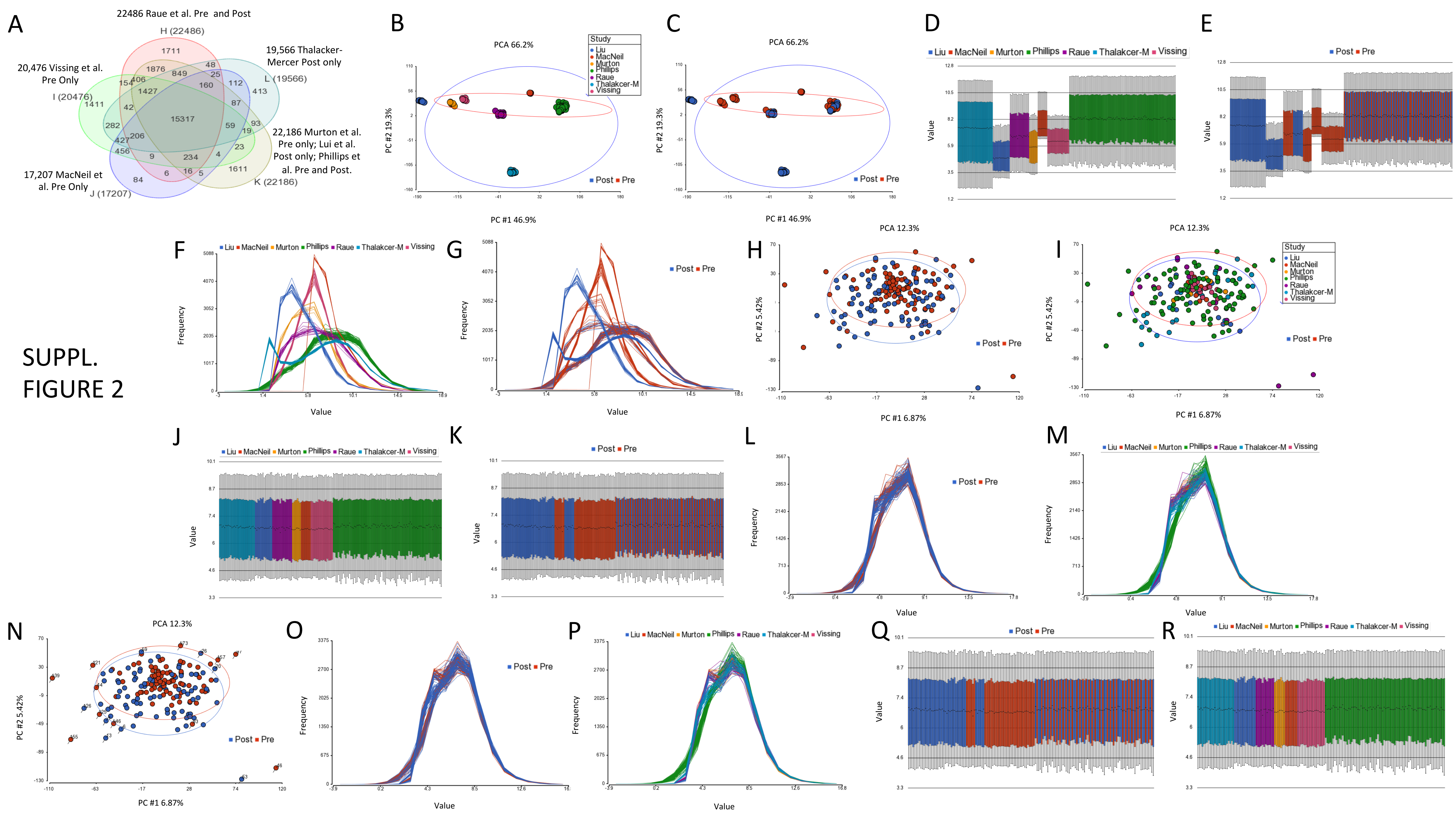

SUPPL. FIGURE 2

Suppl. Figure 2: A, Venn diagram analysis identified a shared list of 15,317 annotated genes by 'gene symbol' across the pooled transcriptomic studies for chronic RE. **B,** PCA for study and **C,** time (pre / post) **D,** box and whisker plots by study and **E,** by time point. **F,** frequency/density plots by lines for study and **G** time (pre/post). There was noticeable signal/ value variation as a result of batch effects across studies. Following batch correction: **H,** PCA by time and **I,** by study demonstrated smaller variation than prior to batch correction (B & C above). **J** Box and whisker plots by study and **K,** by time post batch removal. **L,** Frequency plots by time and **M,** by study post batch removal demonstrated more appropriate signals and distribution compared with those prior to batch removal (D-G). **N,** PCA post batch correction by time (pre/post) depicting the outliers removed (line strikethrough) that were located outside 2SD's of the centroid value using ellipsoids. **O,** Frequency/density plots by time and **P,** by study post batch removal with outliers (identified in N) removed. **Q,** Box and whisker plots by time and **R,** by study post batch removal with outliers (identified in N) removed.
