# Supplementary material for: Comparative Transcriptome and Methylome Analysis in Human Skeletal Muscle Anabolism, Hypertrophy and Epigenetic Memory
Source: Sci Rep. 2019 Mar 12;9:4251. doi: 10.1038/s41598-019-40787-0 (PMC6414679; doi:10.1038/s41598-019-40787-0)
Supplement: Supplementary file 2 — Suppl. File 2 [file 41598_2019_40787_MOESM2_ESM.docx]

**Comparative Transcriptome and Methylome Analysis in Human Skeletal Muscle Anabolism, Hypertrophy and Epigenetic Memory**

Daniel C. Turner ^1, 2#^, Robert A. Seaborne ^1, 2#^, Adam P. Sharples ^1, 2 *^

^1^ Research Institute for Sport and Exercise Sciences, Liverpool John Moores University, Liverpool, United Kingdom.

^2^ Institute for Science and Technology in Medicine (ISTM), School of Medicine, Keele University, Staffordshire, United Kingdom.

# Contributed equally to this work.

* Corresponding/Senior Author

Address for correspondence:

Dr. Adam P. Sharples

Life Sciences Building

Byrom Street, L3 3AF,

Liverpool John Moores University,

Liverpool,

L3 3AF,

United Kingdom

Email: [a.p.sharples@googlemail.com](mailto:a.p.sharples@googlemail.com)

**Table 1**. **Gene expression transcriptome studies pooled for analysis of gene expression for both acute and chronic resistance exercise (RE)**. Note: There were only 2 studies that contained relevant rested baseline samples prior to (pre) chronic RE within the same study, severely limiting the number of pre/rested samples for the chronic RE analysis ^5,9^, whereas a total of 4 studies had rested post chronic resistance exercise samples (^5, 8, 9, 10^ with ^8,10^ without relevant resting baseline/pre samples). We therefore included the samples from the pre-acute RE transcriptome analysis from the three studies of ^3,4,6^ as relevant pre/baseline rested samples.

| **Acute RE Studies**  MacNeil et al. (2010)^3^  Raue et al. (2012)^5^  Vissing & Schjerling. (2014)^6^  Murton et al. (2014)^4^  Lundberg et al. (2016)^7^  **Chronic RE Studies**  MacNeil et al. (2010)^3^  Liu et al. (2010)^8^  Raue et al. (2012)^5^  Phillips et al. (2013)^9^  Thalacker-Mercer et al. (2013)^10^  Vissing & Schjerling. (2014)6  Murton et al. (2014)^4^ | **PMID**  20502695  22302958  25984345  24265280  27101291  20502695  21106073  22302958  23555298  23632419  25984345  24265280 | **GEO Acc.**  GSE19062  GSE28422  GSE59088  GSE45426  GSE74194  GSE19062  GSE24235  GSE28422  GSE47881  GSE42507  GSE59088  GSE45426 | **Array Platform**  GPL6255 Illumina humanRef-8 v2.0  GPL570 [HG U133_Plus_2]  GPL6244 [HuGene-1_0-st]  GPL570 [HG U133_Plus_2]  GPL17692 [HuGene-2_1-st]  GPL6255 Illumina humanRef-8 v2.0  GPL570 [HG U133_Plus_2]  GPL570 [HG U133_Plus_2]  GPL570 [HG U133_Plus_2]  GPL6480 Agilent-014850  GPL6244 [HuGene-1_0-st]  GPL570 [HG U133_Plus_2] | **Exercise**  Eccentric exercise of the quadriceps, 15 sets of 10 reps. maximally resisting flexion of the knee at 120°/s, 1 min rest between sets.  Bilateral knees extension of the quadriceps, 3 sets of 10 reps at 70-75% of their 1RM  3 separate exercises of the quadriceps x 1 set of their 12 RM, 1.5 mins. rest between exercises.  Knee extension, 5 sets of 30 maximal isokinetic contractions at 180°/s, 1 min rest between sets.  Knee extension, 4 sets of 7 reps (70% of max.), 2 min rest between sets.  Pre (rested) samples used in analysis only.  2/wk by 12 wks progressive RE of the arms (5 elbow flexor exercise x 3 sets of 6 RM).  3/wk by 12 wks Bilateral knees extension of the quadriceps, 3 sets of 10 reps at 70-75% of their 1RM.  3/wk by 20 wks progressive RE (4 wks 40-60% 1RM, 16 wks 70% 1RM) 1RM assessed every 4 wks. Multiple sets (no. not described) x 12 reps.  3/wk by 16 wks RE (specific intensity or exercises performed not described)  Pre (rested) samples used in analysis only.  Pre (rested) samples used in analysis only. | **Time point**  Pre, 3h Post  Pre, 4h Post  Pre, 2.5h, 5h Post  Pre, 24h Post  Post 3h Only  Pre Only  Post Only  Pre, Post  Pre, Post  Post Only  Pre Only  Pre-Only | **Notes**  E2 supplement group was removed. Only placebo/no supplement group analysed.  Elderly male/ female and young female adult acute and chronic RE groups were removed. Only young male adult group analysed.  Endurance exercise group was removed. Only pre and post-acute RE were analysed.  Non-exercise group was removed. Pre/post-acute RE group used for the analysis.  No relevant pre, as the comparison was post-acute RE from one limb (that performed RE) vs. the contralateral limb that underwent both RE plus endurance exercise. Post RE limb samples used for the analysis only. RE+ endurance limb samples were removed.  Pre (resting) samples (from acute RE transcriptome analysis above) only were included in the analysis. E2 supplement group was removed. Only placebo/no supplement group analysed.  Analysed the resting samples only from biopsies of the arm that had done the 12 weeks chronic RE.  Elderly male / female and young female adult acute RE groups were removed. Only young male adult group pre and post chronic RE at rest were analysed.  Resting Pre and Post Chronic samples analysed.  Non-responder group was removed. Moderate and extreme responders analysed only.  Pre (resting) samples (from acute RE transcriptome analysis above) were included in the analysis.  Pre (resting) samples (from acute RE transcriptome analysis above) were included in the analysis. |
| --- | --- | --- | --- | --- | --- | --- |
