# Supplementary material for: Comparative Transcriptome and Methylome Analysis in Human Skeletal Muscle Anabolism, Hypertrophy and Epigenetic Memory
Source: Sci Rep. 2019 Mar 12;9:4251. doi: 10.1038/s41598-019-40787-0 (PMC6414679; doi:10.1038/s41598-019-40787-0)
Supplement: Supplementary file 9 — Suppl. File 9 [file 41598_2019_40787_MOESM9_ESM.docx]

**Comparative Transcriptome and Methylome Analysis in Human Skeletal Muscle Anabolism, Hypertrophy and Epigenetic Memory**

Daniel C. Turner ^1, 2#^, Robert A. Seaborne ^1, 2#^, Adam P. Sharples ^1, 2 *^

^1^ Research Institute for Sport and Exercise Sciences, Liverpool John Moores University, Liverpool, United Kingdom.

^2^ Institute for Science and Technology in Medicine (ISTM), School of Medicine, Keele University, Staffordshire, United Kingdom.

# Contributed equally to this work.

* Corresponding/Senior Author

Address for correspondence:

Dr. Adam P. Sharples

Life Sciences Building

Byrom Street, L3 3AF,

Liverpool John Moores University,

Liverpool,

L3 3AF,

United Kingdom

Email: [a.p.sharples@googlemail.com](mailto:a.p.sharples@googlemail.com)

**Suppl. File 8:**

**13 genes upregulated and hypomethylated after acute RE involved in ECM/Actin structure/remodelling and mechanotransduction.**

**MSN** (Meosin) involved in actin and plasma membrane crosslinking and associated with muscle dystrophies ^1^.

**THBS1** (Thrombospondin 1) an adhesive glycoprotein mediating cell-to-cell and matrix interactions that can bind to fibrinogen and as well as ECM proteins such as laminin, collagen and integrins, with a demonstrated role in capillary density in muscle ^2^.

**TIMP3** (Metalloproteinase inhibitor 3) involved in remodelling the matrix ^3^.

**FLNB** (Filamin B) that connects the cell membrane to actin cytoskeleton, and mutations that lead to myopathies ^4^.

**LAMA5** (Laminin 5) an ECM protein that is altered after remodelling of the muscle matrix ^5^;

**CRK** (Adapter molecule crk) regulates cell adhesion associated with mechano-transduction via FAK ^6,7^.

**COL4A1** (Collagen alpha-1 IV chain) is a major structural component of basement membranes, linking to other laminins and proteoglycans^8^.

**ITGA2** (Integrin alpha-2/beta-1) is a receptor for laminin, collagen, fibronectin and E-cadherin.

**ITGB3** (Integrin Beta-3) together with Integrin Alpha 5 is a receptor for ECM proteins in muscle, and important in muscle cell migration ^9^.

**CD63** (CD63 antigen), functions as cell surface receptor for TIMP1^10^ and is involved in the activation of integrin/FAK/AKT signalling.

**CTTN** (Src substrate cortactin) contributes to the organization of the actin cytoskeleton ^11^ and actin filament remodelling in L6 myotubes ^12^.

**F2RL3** (aka PAR-4 Proteinase-activated receptor 4) is a receptor for activated thrombin or trypsin, however it demonstrated no real role the in differentiation in muscle cells ^13^.

**RASSF5** (Ras association domain-containing protein 5 aka. RAPL), although no known role in skeletal muscle, together with RAP1A is involved in extension of microtubules in endothelial cells ^14^.

**14 genes upregulated and hypomethylated after chronic RE involved in ECM/Actin structure/remodelling and mechanotransduction.**

**COL4A2** (Type IV collagen) structural protein in the ECM.

**HSPG2** aka. Perlecan (Basement membrane-specific heparan sulfate proteoglycan core protein). Important component of basement membranes

**ITGA6** (Integrin alpha-6) is a receptor for laminin. ITGA6:ITGB4 binds to IGF-I/2 and this binding is essential for IGF-I/2 signaling.

**TIAM1** (**T-lymphoma invasion and metastasis-inducing protein 1)** that connects extracellular signals to cytoskeletal activities.

**CTTN** (cortactin) contributes to the organization of the actin cytoskeleton.

**GNA12** (Guanine nucleotide-binding proteins-G proteins) involved as transducer in transmembrane signalling.

**ADCY4** (Adenylate cyclase type 4) involved in G-protein signalling.

**BCR** (Breakpoint cluster region protein) is a GTPase-activating protein.

**PTK2 aka. FAK** (Focal adhesion kinase) located in the mechano-sensing costamere of skeletal muscle; PLCG2, an enzyme involved in transmembrane signalling.

**FN1** (Fibronectin) binds cell surfaces including collagen, fibrin, heparin, and actin; PLD-1; (Phospholipase D1) involved in transmembrane trafficking.

**FLNB** (Filamin-B) that connects cell membrane constituents to the actin cytoskeleton.

**PLAUR** (Urokinase plasminogen activator surface receptor) acts as a receptor for urokinase plasminogen activator.

**EZR** (Ezrin) involved in connections of major cytoskeletal structures to the plasma membrane.

**References**

1 Pines, M. *et al.* Elevated Expression of Moesin in Muscular Dystrophies. *Am J Pathol* **187**, 654-664, doi:10.1016/j.ajpath.2016.11.013 (2017).

2 Audet, G. N., Fulks, D., Stricker, J. C. & Olfert, I. M. Chronic delivery of a thrombospondin-1 mimetic decreases skeletal muscle capillarity in mice. *PLoS ONE* **8**, e55953, doi:10.1371/journal.pone.0055953 (2013).

3 Guzzoni, V. *et al.* Effect of Resistance Training on Extracellular Matrix Adaptations in Skeletal Muscle of Older Rats. *Frontiers in Physiology* **9**, 374, doi:10.3389/fphys.2018.00374 (2018).

4 Xu, Q., Wu, N., Cui, L., Wu, Z. & Qiu, G. Filamin B: The next hotspot in skeletal research? *Journal of genetics and genomics = Yi chuan xue bao* **44**, 335-342, doi:10.1016/j.jgg.2017.04.007 (2017).

5 Rayagiri, S. S. *et al.* Basal lamina remodeling at the skeletal muscle stem cell niche mediates stem cell self-renewal. *Nature Communications* **9**, 1075, doi:10.1038/s41467-018-03425-3 (2018).

6 Goel, H. L. & Dey, C. S. PKC-regulated myogenesis is associated with increased tyrosine phosphorylation of FAK, Cas, and paxillin, formation of Cas-CRK complex, and JNK activation. *Differentiation; research in biological diversity* **70**, 257-271, doi:10.1046/j.1432-0436.2002.700604.x (2002).

7 Kawauchi, K. *et al.* p130Cas-dependent actin remodelling regulates myogenic differentiation. *Biochem J* **445**, 323-332, doi:10.1042/bj20112169 (2012).

8 Hyldahl, R. D. *et al.* Extracellular matrix remodeling and its contribution to protective adaptation following lengthening contractions in human muscle. *Faseb j* **29**, 2894-2904, doi:10.1096/fj.14-266668 (2015).

9 Sinanan, A. C., Machell, J. R., Wynne-Hughes, G. T., Hunt, N. P. & Lewis, M. P. Alpha v beta 3 and alpha v beta 5 integrins and their role in muscle precursor cell adhesion. *Biol Cell* **100**, 465-477, doi:10.1042/bc20070115 (2008).

10 Jung, K. K., Liu, X. W., Chirco, R., Fridman, R. & Kim, H. R. Identification of CD63 as a tissue inhibitor of metalloproteinase-1 interacting cell surface protein. *Embo j* **25**, 3934-3942, doi:10.1038/sj.emboj.7601281 (2006).

11 von Holleben, M., Gohla, A., Janssen, K. P., Iritani, B. M. & Beer-Hammer, S. Immunoinhibitory adapter protein Src homology domain 3 lymphocyte protein 2 (SLy2) regulates actin dynamics and B cell spreading. *J Biol Chem* **286**, 13489-13501, doi:10.1074/jbc.M110.155184 (2011).

12 Nazari, H. *et al.* Cortactin, an actin binding protein, regulates GLUT4 translocation via actin filament remodeling. *Biochemistry. Biokhimiia* **76**, 1262-1269, doi:10.1134/s0006297911110083 (2011).

13 Chinni, C. *et al.* Thrombin, a survival factor for cultured myoblasts. *J Biol Chem* **274**, 9169-9174 (1999).

14 Fujita, H. *et al.* Local activation of Rap1 contributes to directional vascular endothelial cell migration accompanied by extension of microtubules on which RAPL, a Rap1-associating molecule, localizes. *J Biol Chem* **280**, 5022-5031, doi:10.1074/jbc.M409701200 (2005).
